# Nasopharyngeal SARS-CoV-2 may not be dispersed by a high-flow nasal cannula

**DOI:** 10.1038/s41598-023-29740-4

**Published:** 2023-02-15

**Authors:** Tetsuya Suzuki, Shinichiro Morioka, Kei Yamamoto, Sho Saito, Shun Iida, Katsuji Teruya, Jin Takasaki, Masayuki Hojo, Kayoko Hayakawa, Satoshi Kutsuna, Sho Miyamoto, Seiya Ozono, Tadaki Suzuki, Eiichi N. Kodama, Norio Ohmagari

**Affiliations:** 1 Disease Control and Prevention Center, National Center for Global Health and Medicine, 1-21-1, Toyama, Shinjuku-Ku, Tokyo, 162-8655 Japan; 2 Emerging and Re-Emerging Infectious Diseases, Graduate School of Medicine, Tohoku University, Sendai, Japan; 3 Department of Pathology, National Institute of Infectious Diseases, Tokyo, Japan; 4 AIDS Clinical Center, National Center for Global Health and Medicine, Tokyo, Japan; 5 Department of Respiratory Medicine, National Center for Global Health and Medicine, Tokyo, Japan; 6 Department of Infection Control, Graduate School of Medicine/Faculty of Medicine, Osaka University, Osaka, Japan; 7 Division of Infectious Disease, International Research Institute of Disaster Science, Graduate School of Medicine, Tohoku Medical Megabank Organization, Tohoku University, Sendai, Japan

**Keywords:** Infectious diseases, SARS-CoV-2, Air microbiology

## Abstract

A high-flow nasal cannula (HFNC) therapy plays a significant role in providing respiratory support to critically ill patients with coronavirus disease 2019 (COVID-19); however, the dispersion of the virus owing to aerosol generation is a matter of concern. This study aimed to evaluate if HFNC disperses the virus into the air. Among patients with COVID-19 admitted to private rooms with controlled negative pressure, we enrolled those admitted within 10 days of onset and requiring oxygenation through a conventional nasal cannula or HFNC therapy. Of the 17 patients enrolled, we obtained 22 samples (11 in the conventional nasal cannula group and 11 in the HFNC group). Viral RNA was detected in 20 nasopharyngeal swabs, and viable viruses were isolated from three nasopharyngeal swabs. Neither viral RNA nor viable virus was detected in the air sample at 0.5 m regardless of the oxygen-supplementation device. We detected viral RNA in two samples in the conventional nasal cannula group but not in the HFNC therapy group in gelatin filters located 3 m from the patient and the surface of the ventilation. This study directly demonstrated that despite viral RNA detection in the nasopharynx, viruses may not be dispersed by HFNC therapy. This warrants further research to determine if similar results can be obtained under different conditions.

## Introduction

The coronavirus disease 2019 (COVID-19) pandemic, which began toward the completion of 2019, is ongoing as of April 2022^1^. It continues to impose a heavy burden on healthcare institutions, related organizations, and citizens worldwide. Nosocomial infections among healthcare workers have been a problem since the early stage of the COVID-19 pandemic^2^. The standardization of personal protective equipment, hand hygiene, and zoning have been thoroughly implemented for its prevention. Nevertheless, the risk of COVID-19 among healthcare workers in hospitals remains reportedly high^3^, and infection-control personnel in each medical institution are struggling to cope with it.

Patients with severe COVID-19 may develop respiratory failure and require ventilator management or extracorporeal membrane oxygenation. The introduction of such invasive procedures increases the number and effort of medical personnel caring for patients, which in turn upsurges the workload. High-flow nasal cannula (HFNC) therapy is a respiratory support device to administer a considerably larger amount of oxygen; it reduces the need for mechanical ventilation in patients with severe COVID-19^4^. However, the administration of high-flow oxygen supposedly generates aerosols, and concerns have been raised about the risk of nosocomial infections^5^. A previous study^6^ reported that HFNC did not increase the risk of infection among healthcare workers; however, the study compared the positivity rate of COVID-19 among healthcare workers before and after the introduction of HFNC therapy. There are insufficient data on the extent of viral dispersion following HFNC use. Therefore, we used air sampling to detect viable severe acute respiratory syndrome coronavirus 2 (SARS-CoV-2) in the air of rooms where patients with COVID-19 receiving oxygen through HFNC or conventional nasal cannula were admitted.

## Results

A total of 17 patients (eight patients in the conventional nasal cannula group and nine patients in the HFNC group) were subjected to 22 samplings (11 samplings in each group). The median age was 59 years (range: 31–86), and the median body mass index was 24.9 kg/m^2^ (range: 16.8–40.8). Among the total patients, 64.7% were men, and 47.1% of the patients had diabetes. All patients had radiological findings of pneumonia and were treated with remdesivir and systemic corticosteroids. Sampling was performed at a median of 8.5 days (range, 2–10) from the onset. The median room temperature and humidity were 24.0 °C and 56.0%, respectively. The patient and sampling background did not significantly differ between the groups (Table 1).

**Table 1:** Characteristics of the patients and samplings.

	Overall	Conventional nasal cannula	High-flow nasal cannula	*p* value
Number of patients	17	8	9	
Age, years	59 [31–86]	61.5 [31–84]	59 [34–86]	0.847
Sex				1.000
Men	11	5 (62.5)	6 (66.7)	
Women	6	3 (37.5)	3 (33.3)	
BMI, kg/m^2^	24.9 [16.8–40.8]	26.4 [16.8–35.0]	24.9 [20.8–40.8]	0.773
COPD	1 (5.88)	1 (12.5)	0	0.471
Asthma	2 (11.8)	2 (25.0)	0	0.206
Diabetes	8 (47.1)	4 (44.4)	5 (55.6)	0.637
Pneumonia	17 (100)	8 (100)	9 (100)	NA
Remdesivir	17 (100)	8 (100)	9 (100)	NA
Systemic steroid	17 (100)	8 (100)	9 (100)	NA
Number of samplings	22	11	11	
Days from onset	8.5 [2–10]	8 [2–10]	9 [2–10]	0.640
Oxygen flow, L/min	NA	2 [1–4]	50 [40–60]	NA
Room temperature, °C	24.0 [23.2–26.0]	24.1 [23.4–26.0]	24.0 [23.2–25.4]	0.337
Room humidity, %	56 [36–74]	55 [37–74]	57 [36–73]	1.000

*BMI* body mass index, *COPD* chronic obstructive pulmonary disease; and *NA* not applicable.

Data are presented as number (%) or median [range].

Table 2 summarizes the results for each sampling. Of the 11 nasopharyngeal samples in the conventional nasal cannula group, viral RNA was detected in nine (81.8%) samples. However, the virus was isolated from only two (18.2%) samples. Of the samples collected from the gelatin filters at 0.5 m and 3 m from the patient and the surface of the ventilation duct, viral RNA was detected in only two samples from the gelatin filter at 3 m in the conventional nasal cannula group; however, we did not isolate a viable virus. The cycle threshold value of the two samples from gelatin filters at 3 m and the corresponding nasopharyngeal swabs were 41.35 and 39.41 and 16.71 and 33.89, respectively. In the HFNC group, viral RNA was detected in all nasopharyngeal samples, but the virus was isolated from only one (9.1%) sample. No viral RNA was detected, and SARS-CoV-2 was not isolated from air samples at 0.5, 3 m, or the surface of the ventilation duct. Patient D was sampled once while on HFNC and once following a transition to the conventional nasal cannula. Data on the background factors of patient D are presented only in the HFNC group. A comparison of the polymerase chain reaction (PCR)-positivity rate in the nasopharyngeal swab between the two groups by an oxygen administration device was statistically insignificant (*p* = 0.476). Further, the detection rate of a viable virus in the nasopharyngeal swab was statistically insignificant (*p* = 1.0). Among patients in whom viral RNA was detected in the nasopharyngeal samples, the cycle threshold value of reverse transcription-quantitative PCR (RT-qPCR) was lower in the conventional nasal cannula group than that in the HFNC group (median cycle threshold value, 24.83 vs. 27.57, *p* = 0.020). The detection rate of viral RNA in the air sample obtained at 3 m was not significantly different between the groups (*p* = 0.190).

**Table 2:** Details of each sample and the results of viral detection.

Device	Patient	Oxygen flow, L/min	Age, years	Days from onset	Variant	Cough during sampling	Nasopharyngeal swab		0.5 m, air		3 m, air		Duct surface	
							RNA*	Isolation	RNA	Isolation	RNA*	Isolation	RNA	Isolation
Conventional nasal cannula	A	1	84	2	Alpha	0	16.71	Positive	–	NE	41.35	–	–	NE
	B	2	68	3	Unknown	0	18.62	Positive	–	NE	–	NE	–	NE
	C	2	51	7	Alpha	1	27.21	–	–	NE	–	NE	–	NE
	D	1	73	7	Alpha	0	33.89	–	–	NE	39.41	–	–	NE
	E	3	57	8	Delta	1	21.75	–	–	NE	–	NE	–	NE
	F	3	66	8	Alpha	0	–	NE	–	NE	–	NE	–	NE
	F	2	66	9	Alpha	0	–	NE	–	NE	–	NE	–	NE
	G	1	31	9	Delta	5	25.04	–	–	NE	–	NE	–	NE
	H	3	81	9	Alpha	1	24.83	–	–	NE	–	NE	–	NE
	H	4	81	10	Alpha	0	25.63	–	–	NE	–	NE	–	NE
	I	1	44	10	Alpha	0	21.96	–	–	NE	–	NE	–	NE
High-flow nasal cannula	J	55	49	2	Alpha	0	35.86	–	–	NE	–	NE	–	NE
	J	50	49	4	Alpha	0	35.91	–	–	NE	–	NE	–	NE
	D	50	73	5	Alpha	0	34.69	–	–	NE	–	NE	–	NE
	K	50	53	7	Delta	0	29.60	–	–	NE	–	NE	-	NE
	L	60	59	8	Unknown	0	25.66	Positive	–	NE	–	NE	–	NE
	M	55	86	9	Alpha	0	23.68	–	–	NE	–	NE	–	NE
	N	40	74	9	Unknown	0	22.89	–	–	NE	–	NE	–	NE
	N	40	74	10	Unknown	0	27.02	–	–	NE	–	NE	–	NE
	O	50	34	10	Alpha	1	29.47	–	–	NE	–	NE	–	NE
	P	40	55	10	Delta	0	27.57	–	–	NE	–	NE	–	NE
	Q	50	67	10	Delta	2	27.05	–	–	NE	–	NE	–	NE

*The cycle threshold value is presented for RNA detection.

*NE* Not examined.

## Discussion

In this study, SARS-CoV-2 was not detected in the air regardless of the flow rate of oxygen administration. Our results would be applied in negative-pressure rooms comprising patients admitted within 10 days of COVID-19 onset. Moreover, viral RNA was not particularly dispersed in the air via HFNC therapy, despite its presence in the nasopharynx. In a previous study evaluating the dispersal of droplets and aerosols by HFNC therapy and conventional nasal cannulas, HFNC therapy did not increase nosocomial SARS-CoV-2 infection among healthcare workers^7^. In our study, we directly demonstrated that nasopharyngeal viruses were not dispersed by HFNC therapy by collecting air samples from rooms with hospitalized patients.

Viral RNA was detected in no air samples collected at 0.5 m from the patients and in a few samples collected at 3 m. COVID-19 can be transmitted through aerosols drifting through the air beyond the range of droplet infection^8^. In contrast, larger droplets settle down within a few seconds to a few minutes, and the role of infection by inhalation increases with the distance^9^, partially supported by our results. At 0.5 m, virus-containing droplets may have settled onto the surface of the desk or floor; therefore, air sampling did not capture these droplets. At 3 m, virus-containing aerosols were drifting in the air and were captured during air sampling. In addition, the position of the duct in the room and the air sampler may have been influenced. Because the duct was located above the patient's head, the air sampler placed at a distance of 0.5 m was closer to the duct. The difference in room ventilation efficiency owing to the position of the duct may have resulted in a variance in the detection rate of virus particles in the air samples. Advanced computer simulation studies would be required to confirm this hypothesis; however, we were unable to perform such an analysis.

At 3 m, we detected viral RNA only in the conventional nasal cannula group. The median cycle threshold value of the nasopharyngeal swab was lower in the conventional nasal cannula group (with a greater amount of viral particles) than that in the HFNC group. This difference in the number of viral particles in the nasopharynx between the groups may have influenced this result; however, the rate of viral detection at 3 m was not significantly different between the groups. In addition, we isolated limited infectious viruses, despite detecting viral RNA in the nasopharynx. In a previous study of patients with COVID-19 without oxygen supplementation, the viral isolation rate at 0–5 days and 6–9 days following symptom onset was 27.3 and 30.8%, respectively^10^. In our study, infectious viruses were isolated in only 13.6% of the overall samples. The amount of SARS-CoV-2 in the nasopharynx of patients with COVID-19 is the highest before and immediately following the onset of the disease^11^. In contrast, pneumonia and increased oxygen demand are supposedly caused by subsequent excessive inflammation, characterized by lower viral load^12^. Thus, patients with COVID-19, in whom the disease has progressed to pneumonia, may exhibit fewer viral particles and be considerably less infectious. Our findings suggested that HFNC therapy may result in a similar extent of SARS-CoV-2 dispersal in the air and overall infection risk compared with those upon using the conventional nasal cannula.

Further, our air sampling technique may have influenced the results. The method of air sampling has been validated by a previous study^13^. However, our sampling conditions were different from those in the mentioned study. In addition, owing to the structure of the room and the location of the ventilation duct, the ventilation efficiency around the air sampler may have differed even within an identical room. Furthermore, the method of assessing the risk of infection using air sampling techniques is still under development. Measles virus, an airborne virus, was detected by air sampling at 8 feet from the patient^14^. Conversely, the influenza virus, which does not cause airborne transmission, was detected by air sampling at 2.2 m from the patient^15^. Further accumulation of scientific findings is required to accurately determine the possibility of airborne transmission of a disease by air sampling.

This study had certain limitations. First, the sample size was small, and further studies are required to accumulate robust scientific evidence. We performed a limited evaluation of the quantitative infection risk during HFNC therapy because infectious viruses were not detected in the majority of the nasopharynx. However, the low detection of infectious viruses during pneumonia suggested a low risk of nosocomial infection in clinical settings. Second, the study was conducted in negative-pressure private rooms; thus, the results should be cautiously applied to rooms without pressure control. Generally, negative pressure can be managed in a limited number of private rooms. Moreover, it is crucial to avoid intubation by using HFNC to reduce the medical burden. Thus, researchers should perform similar studies to investigate the safety of HFNC in rooms without negative pressure. Third, virus dispersal may change with the severity of the illness evaluated and classified by other criteria. In our study, we did not include the body temperature, inflammatory markers (e.g., C-reactive protein), or the extent of radiological findings of pneumonia. The amount of oxygen supplement and oxygen delivery devices presumably reflect the severity of COVID-19; nonetheless, severity based on other conditions may yield different results. Thus, exploratory studies are warranted to identify the factors related to virus dispersal; however, we did not perform such an analysis because of the small sample size. Furthermore, this study was conducted before the prevalence of the omicron strain, and the results may change in the future depending on viral mutations.

In conclusion, we detected limited viral RNA in the air in negative-pressure private rooms comprising patients with COVID-19 admitted for HFNC therapy. Furthermore, the infectious virus may not be dispersed in the air, despite detecting viral RNA in the nasopharynx. To downscale infection control in actual clinical settings, further research is required to determine if similar results can be obtained under various room conditions in terms of pressure control and room size and with mutant strains that may emerge in the future.

## Methods

### Study design, setting, and population

This cross-sectional study included patients with confirmed COVID-19 who were admitted to the National Center for Global Health and Medicine (NCGM) between April 28, 2021, and August 12, 2021. Among those admitted to private rooms with controlled negative pressure, we enrolled patients who were admitted within 10 days of COVID-19 onset and required oxygenation through a conventional nasal cannula or HFNC. The limit of 10 days was set to allow the detection of infectious viruses from the nasopharynx. The rooms were designed to have air changes at least thrice per hour. Epidemiological and relevant data, including comorbidities and the treatment for COVID-19, were obtained from the medical records. Written informed consent was obtained from all participants. This study was approved by the Institutional Review Board of National Center for Global Health and Medicine (approval number: NCGM-G-004132). All methods were performed in accordance with the relevant guidelines and regulations.

### Sample collection

Air sampling was performed using an AirPort MD8 air sampler (Sartorius, Goettingen, Germany) with sterile gelatin filters (80-mm diameter and 3-µm pores; Sartorius). We set one device on the over-bed table (0.5 m from the patient, within the droplet infection range) and another device on a chair 3-m from the patient (beyond the droplet infection range) for simultaneous air sampling. The room ventilation duct was located above their heads. The flow rate was 125 L/min, and we aspirated a total volume of up to 2,000 L. The filters were rapidly and aseptically dissolved in 12 mL of the viral transport medium (Sugiyamagen, SGVTM-3R). The surfaces of the room ventilation duct on the ceiling were wiped thrice using a swab moistened with a medium at different locations, and these swabs were suspended in 3 mL of a viral transport medium. Further, nasopharyngeal swabs were collected and suspended in 3 mL of this medium. All specimens were frozen at -80 °C until the analysis. Specimen collection was repeated every few days until the patient deviated from the inclusion criteria (e.g., > 10 days following the onset of illness, transferred from a private room, no longer required oxygen, or placed on ventilator management). During the air sampling, we measured the temperature, humidity, and cough. During the sampling, the patients removed their surgical masks.

### RT-qPCR sample analysis

Total nucleic acid was extracted from 200 μL of each sample using the KingFisher Apex System (Thermo Fisher Scientific, MA, USA) with the MagMAX Viral/Pathogen Nucleic Acid Isolation Kit (Thermo Fisher Scientific). To detect SARS-CoV-2 RNA from 5 μL of the total nucleic acid samples, we performed an RT-qPCR analysis using the QuantiTect Probe RT-PCR Kit (Qiagen, Hilden, Germany), with the N2 primer/probe set as previously described^16^.

### Viral isolation

We performed viral isolation for all RT-qPCR-positive cases with the available residual specimens as described previously^17^. Briefly, VeroE6/TMPRSS2 cells were seeded in 96-well flat-bottom plates; 100 μL of respiratory and air samples were mixed with Dulbecco’s Modified Eagle Medium supplemented with 2% bovine fetal serum and an antibiotic–antimycotic solution (Thermo Fisher Scientific); they were inoculated in duplicates. The culture supernatant was changed to a fresh medium 1 day post-infection (d.p.i.) and incubated at 37 °C with 5% CO_2_. On 5 d.p.i., we observed a cytopathic effect. Following 5 d.p.i., the supernatant was collected, and RT-qPCR was performed using the SARS-CoV-2 direct detection RT-qPCR kit (Takara Bio, Shiga, Japan) to confirm the propagation of SARS-CoV-2.

### Statistical analyses

To investigate if the HFNC dispersed the virus, the patients were classified into two groups according to the oxygen supplemental device, namely the conventional nasal cannula group and the HFNC group. This classification directly reflected the severity of COVID-19. Samples collected from similar patients were handled as independent data. This is because their nasopharyngeal specimens and relevant environmental data were collected each time. Continuous variables are expressed as medians and ranges, and categorical variables are expressed as numbers and percentages. We performed a two-tailed Fisher’s exact probability test or Mann–Whitney U test to assess differences between the groups, as appropriate. All statistical analyses were performed using R version 4.1.3 (https://cran.r-project.org/bin/windows/base/old/4.1.3/). Statistical significance was set at *p* < 0.05.

## Data Availability

The data that support the findings of this study shall be made available from the corresponding author upon reasonable request.
